# The Impact of Low Back Pain on the Self-Assessed Health-Related Quality of Life in Colostomy Patients—A Cross-Sectional Study

**DOI:** 10.3390/jcm15124615

**Published:** 2026-06-14

**Authors:** Magdalena Tarkowska, Iwona Głowacka-Mrotek, Bartosz Brzoszczyk, Piotr Jarzemski

**Affiliations:** 1 Department of Urology, Nicolaus Copernicus University in Toruń, Collegium Medicum in Bydgoszcz, 85-094 Bydgoszcz, Poland; bartosz.brzoszczyk@cm.umk.pl (B.B.); piotr.jarzemski@cm.umk.pl (P.J.); 2 Department of Rehabilitation, Nicolaus Copernicus University in Toruń, Collegium Medicum in Bydgoszcz, 85-094 Bydgoszcz, Poland; iwona.glowacka@cm.umk.pl

**Keywords:** colostomy, colorectal cancer, pain, lumbar spine, quality of life, complications

## Abstract

**Introduction:** Low back pain is one of the commonly overlooked late complications of an intestinal stoma. Its severity may be associated with impaired quality of life across multiple dimensions of patient functioning. **Objective:** This cross-sectional study evaluated the impact of low back pain on self-reported health-related quality of life in colostomy patients. **Material and Methods:** The study was conducted using a cross-sectional questionnaire-based design across 12 regional branches of the Pol-ILKO Association in Poland between December 2023 and September 2024. The study sample consisted of 95 patients. The standardized Oswestry Disability Index (ODI) questionnaire, which assesses the level of disability in patients with back pain, and the WHOQOL-BREF questionnaire, which assesses health-related quality of life, were used in the survey. In addition, detailed data on medical history, past surgical interventions, and stoma self-care skills were collected using an author-developed tool. **Results:** Greater disability due to back pain is associated with lower self-rated quality of life. The higher the degree of disability as assessed by the Oswestry questionnaire and the higher the number of postoperative complications, the worse the subjective rating of health-related quality of life (HRQoL) (*p* < 0.05). Factors associated with a significantly (*p* < 0.05) increased risk of lower back pain include postoperative complications, irrespective of the time since stoma creation, as well as avoidance or restriction of full trunk movements. Preoperative agreement on the stoma site was associated with greater independence in stoma hygiene. **Conclusions:** The results underscore the importance of early and targeted interventions to improve physical and psychosocial well-being in the subject population, especially at the preoperative stage. More attention should be paid to the needs of colostomy patients, both in hospitals and in outpatient specialty care centers, to improve their overall quality of life and self-assessment of their condition.

## 1. Introduction

The increasing incidence of colorectal cancer (CRC) is correlated with the level of social development and the adoption of Western lifestyles [1]. CRC is now the third most commonly diagnosed malignancy worldwide [2]. The first-line treatment consists of surgery preceded by neoadjuvant treatment and/or followed by adjuvant treatment [3]. Clinical data indicate a number of benefits of minimally invasive laparoscopic techniques, including shorter hospitalization times, fewer complications and blood loss, and faster return to preoperative functional status, among others [4]. In addition, these benefits often translate into improved long-term postoperative quality of life of patients, especially within the physical health and social dimensions [5,6]. On the other hand, the advantages of the open method include lower costs, the ability of the procedure being carried out by most surgeons, and the accessibility of the entire abdominal cavity. However, the requirement for the incision of the skin, fascia, and the rectus abdominis and oblique muscles increases the rate of surgical site infections, prolongs hospitalization, and temporarily limits the patient’s full mobility, which weakens the muscle tone and strength and increases the risk of postoperative complications, even years after surgery [7]. It also delays the patient’s return to social and professional activities [5]. Depending on the clinical case, surgery may involve the need for a temporary or permanent colostomy, which also results in a change in daily habits related to hygiene, nutrition, and physical activity [8,9]. Permanent colostomy is generally considered less favorable for postoperative quality of life; however, sphincter-sparing surgical interventions are also associated with a number of functional problems [10,11].

Low back pain is one of the commonly overlooked late complications of intestinal stoma, its severity being another factor responsible for the impaired quality of life across multiple dimensions of patient functioning. In some patients, the presence of a colostomy results in a reflexive adoption of a forced posture, which promotes the development or worsening of postural defects and spinal pain [12]. Postoperative complications affecting patient mobility generate the need for additional outpatient stoma care [13]. In addition, research shows that unfavorable stoma location is one of the leading risk factors for complications in the form of delayed recovery [13], and that preoperative agreement regarding the potential stoma site can significantly reduce the risk of mechanical trauma and lower body image assessments [7]. The objective of this cross-sectional study was to evaluate the incidence of low back pain and its impact on the self-assessed health-related quality of life in colostomy patients.

## 2. Material and Methods

This cross-sectional study was carried out in 95 Polish colostomy patients affiliated with Pol-ILKO patient support groups who had met the inclusion criteria listed below and who completely and independently answered the questions provided in the survey instruments. The study was conducted in accordance with the principles of the Declaration of Helsinki and had been approved by the Bioethics Committee of Nicolaus Copernicus University in Toruń (decision no. 479/2023). The survey was conducted in 12 of Pol-ILKO’s 18 regional branches between December 2023 and September 2024. The eligibility for the study was based on the following inclusion and exclusion criteria:

### 2.1. Inclusion Criteria

- Colostomy in place for a minimum of 24 months;
- Willingness to participate in the study;
- Colostomy due to colorectal cancer;
- Membership in the Pol-ILKO Association;
- Overall good health (ECOG performance status 0–1);
- Age over 18 years.

### 2.2. Exclusion Criteria

- Previous restoration of gastrointestinal continuity;
- Presence of comorbid spinal conditions or conditions that significantly affected functioning, i.e., Parkinson’s disease, disability requiring the use of rehabilitation crutches or a wheelchair, status post amputation of an upper or lower limb;
- Grade III obesity;
- Other stomas;
- Active cancer;
- Inability to independently complete the survey tools.

The study was conducted using the following instruments:

The standardized Oswestry Disability Index (ODI) questionnaire, which assesses the level of disability of patients with back pain. The questionnaire consists of 10 domains, two of which assess pain and eight of which assess everyday functioning, i.e., lifting, sitting, sleeping, traveling, personal care, walking, standing, and social activities. The subjects provide their answers using a 6-point scale from 0 to 5. The total score ranges from 0 to 50 points, or 0 to 100%, and facilitates the determination of the degree of disability due to back pain. The results are interpreted as follows: 0–4 points—no disability; 5–14 points—mild disability; 15–24 points—moderate disability; 25–34 points—severe disability; and 35 or more points—extreme disability. The ODI questionnaire is considered the gold standard in the assessment of pain and functional status in patients with spinal pain syndromes.

The WHOQOL-BREF questionnaire—a standardized tool dedicated to self-assessment of health-related quality of life (HRQoL). The questionnaire consists of 26 questions and measures the quality of life in physical, psychological, environmental, and social dimensions. In addition, it includes 2 questions related to health assessment and global perception of the quality of life. Only the results of the global QoL scale were taken into account in this study.

A proprietary survey questionnaire aimed at assessing demographic data, i.e., age, educational background, area of residence, employment, and marital status, as well as data on medical history, past surgical interventions, post-surgical complications, physical activity, current health status, and use of health care facilities.

### 2.3. Statistical Analyses

Statistical analyses were performed in Python (version 3.14) [14] using the pandas [15] (version 2.3.3), NumPy [16] (version 2.4.1), SciPy [17] (version 1.17.0), scikit-posthocs [18] (version 0.11.4), matplotlib [19] (version 3.10.8), and tableone [20] (version 0.9.5) libraries. Student’s *t*-test for independent samples, the Mann–Whitney U-test, the chi-squared test of independence, Fisher’s exact test, a one-sample proportion test, the Kruskal–Wallis test, and Dunn’s test with Bonferroni correction were used, as appropriate, to demonstrate the statistical significance of the relationship between patient profile and the Oswestry Disability Index (ODI) questionnaire scores and sex-related differences accross individual variables.

In addition to basic descriptive statistics, an analysis of the impact of demographic and medical data on the results of the Oswestry questionnaire was also performed. Qualitative scales were used to assess the results of individual questions in the Oswestry questionnaire, while a quantitative scale was used for the overall Oswestry questionnaire score. In addition, the strength of the correlations between variables was examined by calculation of Pearson’s and Spearman’s correlation coefficients along with the respective coefficient significance tests. An ordinal regression model was built to examine the impact of variables on quality of life.

A two-sided significance level of 0.05 was used. Significant differences, that is, differences with *p*-values lower than the adopted significance level, are highlighted in green in the tables.

## 3. Results

The study sample was characterized in terms of demographics (Table 1) and clinical data (Table 2). No statistically significant differences were observed accross the demographic variables analyzed. The analysis revealed that most patients resided in urban areas (77.9%), were professionally inactive (72.6%), and of medium economic status (51.6%). The percentages of men and women were similar. The subgroups did not differ significantly, as confirmed by the statistical tests performed.

Statistical analysis showed that the vast majority (90%) of patients self-managed their stoma, with the percentage of women (96.4%) being significantly (*p* = 0.01) higher than that of men (79.5%). The average time since stoma creation was 13 years. The most common post-surgical complication, irrespective of the time having elapsed since stoma creation, was contact dermatitis around the stoma site. Complications were reported in 31.6% of patients. The stoma site had been selected by the surgeon for about one-half (48%) of the patients. About three-quarters of the patients had undergone the surgery using the open method. About 20% of the study sample avoided full-range trunk movements due to the stoma.

The next stage of the analysis focused on presenting the impact of demographic and clinical data on the prevalence and intensity of low back pain as assessed using the Oswestry questionnaire. Statistical analysis revealed that complications occurring irrespective of the time since stoma creation had the greatest impact on ODI questionnaire scores (*p* < 0.05). Statistically significant (*p* < 0.05) differences were also observed for the number of complications. In patients with 0 or 1 complication, the mean scores on the pain intensity scale were 1.53 and 1.20, respectively. On the other hand, patients with 2 or 3 complications reported mean pain intensity scores of 2.84 and 3.69, respectively. A significant increase in pain intensity scores by a factor as high as 1.63 was observed with the number of complications changing from 1 to 2. A similar effect was noted for the change in pain intensity scale. The average scores reported by patients with 0, 1, 2, and 3 complications amounted to 1.06, 0.95, 2.76, and 3.23, respectively. For patients with 0 or 1 complication, the mean change in pain intensity scores remained more or less at the same level of 1. For 2 complications, an increase to 2.76 (up by 1.81) was observed.

Another variable with a significant (*p* < 0.05) effect on the occurrence and intensity of back pain was the “avoidance of full trunk movements”; it was shown to have a significant effect on the score on 8 out of 10 scales of the ODI questionnaire. Patients who reported restricting full trunk movements due to the stoma presented with an average pain intensity score of 3.26. In contrast, patients providing negative responses presented with an average score of 1.59. An equally large change was observed in the change in pain intensity score. When full trunk movements were restricted or avoided, the average change in pain intensity score increased from 1.14 to 3.26.

Of the demographic variables, the greatest impact on the occurrence and/or severity of back pain was observed for professional activity, which was associated with significantly higher scores (*p*-value) for pain intensity (0.01), sleeping (0.04), traveling (0.01), and change in pain intensity (0.00) scales. Detailed data are presented in Table 3.

The next part of the analysis involved the evaluation of the impact of perceived back pain, as assessed by the Oswestry questionnaire, on the health-related quality of life (HRQoL) as self-assessed by the study patients using the WHOQOL-BREF questionnaire. The Oswestry scores were negatively correlated with the quality-of-life results. This means that the higher the Oswestry questionnaire scores, i.e., the greater severity of pain, the lower the quality of life. The number of complications had by far the greatest impact on the quality of life, with a Spearman correlation coefficient of −0.78. Higher scores for change in pain intensity (–0.56 (<0.001)), pain intensity (–0.54 (<0.001)), sleeping (–0.24 (0.01)), traveling (–0.40 (<0.001)), personal care (–0.24 (0.01)), and the total ODI score (–0.44 (<0.001)) were also significantly (*p* < 0.05) related to patients’ quality of life. No significant correlations were observed between the quality of life and the remaining questionnaire domains. Detailed data are presented in Table 4.

Statistical analysis was also directed at assessing the impact of disability on the self-assessed quality of life. Health-related quality of life as assessed using the WHOQOL-BREF questionnaire was shown to significantly (*p* < 0.05) differ between patients with no disabilities and patients with moderate to severe disability. Detailed data are provided in Table 5.

## 4. Discussion

The study analyzed the impact of back pain on the self-assessed health-related quality of life in patients with colostomy secondary to colorectal cancer. The study group consisted of 95 patients in whom at least 24 months had elapsed since stoma creation. The average time since stoma creation was 13 years. Subjects whose low back pain complaints may have been due to other comorbidities were excluded from the study. The degree of disability was assessed using the standardized Oswestry Disability Index (ODI) questionnaire, which assesses the level of disability of patients with back pain. The quality of life was assessed using the WHOQOL-BREF questionnaire used for self-assessment of health-related quality of life. In addition, a proprietary questionnaire was constructed to gather information on the subjects’ medical history and physical activity habits. The analysis revealed that the factors that could potentially affect the occurrence and/or severity of low back pain in colostomy patients include the presence and number of postoperative complications, as well as significant restriction of full-range trunk movements (e.g., trunk twisting, extending). Other factors with a direct relationship to self-reported pain in this subpopulation of patients included the stoma location being discussed with the surgeon prior to surgery, the independence in hygiene activities, as well as selected demographic variables. In addition, the severity of spinal pain was found to significantly reduce the quality of life of colostomy patients, which was most significantly affected by the number of complications and the degree of disability.

The exercise intolerance in patients undergoing anticancer treatment may be multifactorial in its etiology [21]. Postoperative complications that limit spinal mobility and range of motion in stoma patients are one of the contributing factors. As reported by Parmar et al. in their study of 192 intestinal stoma patients, the significant risk factors for postoperative complications included type of stoma (colostomy) (*p* < 0.05), short length of the stoma (*p* = 0.006), higher BMI (*p* = 0.043), emergency surgery (*p* = 0.002), and lack of preoperative site marking (*p* < 0.001). Problematic stomas were associated with longer hospital stays (*p* < 0.001) and more frequent demand for community care (*p* < 0.001) [13]. Similar results were reported by other researchers, who also highlighted the role of body mass index and emergency surgery as predisposing factors for complications, irrespective of the time since surgery [22]. These findings are in line with the results of the presented study, where complications occurring irrespective of the time since surgery, as well as the number of these complications, had a statistically significant effect on the prevalence and severity of low back pain. In addition, the predisposing factors for the aforementioned complaints included the behaviors associated with deliberate restriction of full-range trunk movements, e.g., trunk twists or trunk extension, which may be related to patients’ fear of stoma bag dislodgement or uncontrolled leakage of intestinal contents or gases [12,23].

Interesting results in this aspect are provided by a prospective non-randomized study involving 72 patients operated on for rectal cancer. The objective of the study was to evaluate spinal mobility and pain depending on the surgical technique (laparoscopy vs. open method) and the type of surgical procedure (abdominoperineal resection vs. laparoscopic anterior resection vs. open anterior resection). The assessments were made prior to the surgery, as well as 3 and 6 months thereafter. Three months after the surgery, a decrease in spinal joint mobility in flexion, extension, and lateral flexion, as well as a decrease in the strength of the rectus abdominis and oblique muscles (*p* < 0.05), was observed in all study groups. In the comparison between the groups, the lowest values, suggesting the greatest limitation of the range of motion, were observed in the abdominoperineal resection group [12]. In our study, colostomy patients also declared consciously restricting full-range trunk movements even years after surgery. In another study, ten months after the completion of cancer treatment, patients having undergone partial colon resection reported significantly higher levels of low back pain (*p* = 0.003) compared to a matched control group. Reduced motor activity in patients treated for colon cancer may be due to the weakening of abdominal press muscles as a result of extensive abdominal incisions. The weakened muscle tone may be related to hyperalgesia as observed in this group of patients [23]. This may help explain the findings of the present study, where up to 75% of the study cohort had undergone open surgeries. Low back and pelvic pain syndromes can result from damage to nerve fibers, as occurring during abdominal surgery or from prolonged immobilization [24]. As suggested by findings from other studies, too much immobilization or reduced activity of the area affected by surgical intervention contributes to a decrease in the thickness of the abdominal press muscles and the weakening of the musculoskeletal apparatus [25]. Changes in abdominal press muscles can negatively affect lumbopelvic stability in patients treated for colorectal cancer [12,23], and are reflected in the subjective assessments of health-related quality of life [26].

Studies by other authors highlighted the benefits of the potential stoma site being determined preoperatively with the patient [13]. Similar results were observed in the presented study, where no relevant conversation with the surgeon was associated with a statistically significant increase in the change in pain intensity scores. This may have been due to the increased risk of mechanical trauma at a suboptimally selected stoma site. Of no small importance in this aspect is perioperative nursing education [27], which significantly improves indicators as used in the assessment of patients’ ability to self-care for their stomas and reduces the risk of complications. The patient’s assumption of responsibility for self-care [9] and the preoperative quality of life are important indicators of postoperative outcomes [28]. No other data are indicative of the relationship between preoperative determination of the stoma site and the incidence of complications [22].

Numerous research studies show that health-related quality of life affects the future outcomes of cancer patients [6,11,29]. The discomfort associated with the colostomy, the change in bowel movements, the unpleasant odor, or the lack of ability and/or opportunity to self-manage a stoma can directly affect not only physical functioning, but also occupational or social roles of patients [6]. Sometimes, a forced posture adopted due to the patient’s fear of compromising/damaging the integrity of the stoma bag can translate into low back pain many years later, another factor that impairs daily functioning.

Multimorbidity is a common problem in patients undergoing anticancer treatment [30]. In this study, the degree of physical disability and the number of postoperative complications were shown to significantly correlate with patients’ subjective assessment of quality of life. The greater the degree of disability, the worse the quality-of-life scores; preoperative agreement on the potential site for stoma creation enhances postoperative independence and may positively affect quality of life, as confirmed by other reports [13].

Of note is the very small number of scientific reports on the incidence of pain and spinal mobility limitations in patients with colostomies performed due to colon cancer, and on the impact of both of these aspects on patients’ quality of life. Importantly, studies were carried out both in patients having undergone surgeries up to a few years earlier and in patients having had many years of experience of living with the stoma after their surgery. As a result, self-assessments of the studied variables could be obtained from patients no longer attending standard outpatient follow-up visits. The narrow inclusion criteria eliminated the influence of uncontrolled factors, i.e., concomitant diseases, on the results of the study. Despite the high cognitive value, the study is fraught with certain limitations. Firstly, although strong correlations have been found, the cross-sectional character of the study makes it impossible to definitively prove any causal relationship, for example, whether the back pain developed as a result of stoma creation or whether it had preceded the surgery. Moreover, it would make sense to expand the research methodology to include, in addition to self-assessment tools, clinically objective measurements of spinal mobility. Secondly, the time since stoma creation significantly differed in study participants, translating to a highly heterogeneous cohort. Thirdly, due to the fact that the participants had been recruited from among members of patient support groups, the sample limits external validity due to the non-representative character of the sample, which translates to limited generalizability of results to all colostomy patients. Therefore, it seems reasonable that research on the subject be continued once the research methodology is refined.

Conducting and publishing similar studies may provide important information to help understand chronic pain processes and limited physical activity in colorectal cancer survivors [23]; quality of life should not be treated as an isolated concept, but rather as one of the key outcomes of cancer treatment [10,11].

## 5. Conclusions

1. The presence of complications, regardless of time since colostomy creation, as well as the number of complications, is associated with a significantly increased risk of occurrence and/or severity of lower back pain.
2. Avoidance or marked restriction of full trunk movements in colostomy patients is associated with a significantly increased risk of back pain and greater pain severity.
3. The higher the intensity of lower back pain and the greater the number of postoperative complications, the lower the self-assessed quality of life of colostomy patients.
4. Preoperative agreement on the planned stoma site may protect against mechanical trauma and may improve stoma self-management.
5. The higher the degree of disability, the lower the self-assessed global quality of life.
6. The surgical method was not significantly associated with the presence of low back pain many years after the procedure.

## Figures and Tables

**Table 1:** Demographic and anthropometric characteristics of the study cohort.

Independent Variable	Value	Total (N = 95)	Women (N = 56)	Men (N = 39)	Test	*p*-Value
Age, mean (SD)		69.5 (9.1)	69.5 (9.2)	69.6 (9.2)	Student’s t-test	0.92
BMI, mean (SD)		27.7 (4.7)	27.2 (4.5)	28.4 (5.0)	Student’s t-test	0.26
Educational background, n (%)	Elementary	7 (7.4)	3 (5.4)	4 (10.3)	Fisher’s test	0.40
	Vocational	28 (29.5)	15 (26.8)	13 (33.3)		
	Secondary	41 (43.2)	28 (50.0)	13 (33.3)		
	Higher	19 (20.0)	10 (17.9)	9 (23.1)		
Area of residence, n (%)	Rural	21 (22.1)	10 (17.9)	11 (28.2)	Chi-squared test	0.34
	Urban	74 (77.9)	46 (82.1)	28 (71.8)		
Employment status, n (%)	Professionally active	26 (27.4)	14 (25.0)	12 (30.8)	Chi-squared test	0.69
	Professionally inactive	69 (72.6)	42 (75.0)	27 (69.2)		
Marital status, n (%)	In a relationship	48 (50.5)	27 (48.2)	21 (53.8)	Chi-squared test	0.74
	Single	47 (49.5)	29 (51.8)	18 (46.2)		
Socioeconomic status, n (%)	Low	10 (10.5)	6 (10.7)	4 (10.3)	Fisher’s test	0.88
	Medium	49 (51.6)	28 (50.0)	21 (53.8)		
	Good	31 (32.6)	18 (32.1)	13 (33.3)		
	Very good	5 (5.3)	4 (7.1)	1 (2.6)		
Household status, n (%)	Living with family	57 (60.0)	30 (53.6)	27 (69.2)	Chi-squared test	0.18
	Living alone	38 (40.0)	26 (46.4)	12 (30.8)		

N—sample size; *p*—statistical significance, SD—standard deviation.

**Table 2:** Medical characteristics of the study cohort.

Independent Variable	Value	Total (N = 95)	Women (N = 56)	Men (N = 39)	Test	*p*-Value
**Time since stoma creation, mean (SD)**		13.6 (7.7)	13.7 (7.5)	13.5 (8.1)	Mann–Whitney U-test	0.94
**Self-management of stoma, n (%)**	**Yes**	85 (89.5)	54 (96.4)	31 (79.5)	Fisher’s test	0.01
	**No**	10 (10.5)	2 (3.6)	8 (20.5)		
**Stoma site decided upon with the surgeon in advance, n (%)**	**Yes**	46 (48.4)	27 (48.2)	19 (48.7)	Chi-squared test	1.00
	**No**	49 (51.6)	29 (51.8)	20 (51.3)		
**Avoidance of full trunk movements, such as trunk twists or extension, n (%)**	**Yes**	19 (20.0)	12 (21.4)	7 (17.9)	Chi-squared test	0.87
	**No**	76 (80.0)	44 (78.6)	32 (82.1)		
**Surgical method, n (%)**	**Laparoscopic**	24 (25.3)	18 (32.1)	6 (15.4)	Chi-squared test	0.10
	**Open**	71 (74.7)	38 (67.9)	33 (84.6)		
**Contact dermatitis around stoma site, n (%)**	**Yes**	30 (31.6)	19 (33.9)	11 (28.2)	Chi-squared test	0.71
	**No**	65 (68.4)	37 (66.1)	28 (71.8)		
**Allergic dermatitis, n (%)**	**Yes**	18 (18.9)	11 (19.6)	7 (17.9)	Chi-squared test	1.00
	**No**	77 (81.1)	45 (80.4)	32 (82.1)		
**Fungal infections of skin around the stoma site, n (%)**	**Yes**	10 (10.5)	6 (10.7)	4 (10.3)	Fisher’s test	1.00
	**No**	85 (89.5)	50 (89.3)	35 (89.7)		
**Pyoderma gangrenosum, n (%)**	**Yes**	3 (3.2)	3 (5.4)	0 (0.0)	Fisher’s test	0.26
	**No**	92 (96.8)	53 (94.6)	39 (100.0)		
**Deep ulcerations of skin around the stoma site, n (%)**	**Yes**	2 (2.1)	1 (1.8)	1 (2.6)	Fisher’s test	1.00
	**No**	93 (97.9)	55 (98.2)	38 (97.4)		
**Bowel obstruction, n (%)**	**Yes**	16 (16.8)	13 (23.2)	3 (7.7)	Chi-squared test	0.08
	**No**	79 (83.2)	43 (76.8)	36 (92.3)		
**Mechanical injuries, n (%)**	**Yes**	10 (10.5)	5 (8.9)	5 (12.8)	Fisher’s test	0.73
	**No**	85 (89.5)	51 (91.1)	34 (87.2)		

N—sample size; *p*—statistical significance, SD—standard deviation.

**Table 3:** Impact of demographic and medical variables on the ODI questionnaire results—qualitative variables.

	Pain Intensity	Lifting	Sitting	Sleeping	Traveling	Personal Care	Walking	Standing	Social Life	Change in Pain Intensity
Educational background	0.07	0.95	0.08	0.56	0.51	0.29	0.00	0.91	0.15	0.63
Area of residence	0.01	0.95	0.09	0.50	0.64	0.46	0.44	0.39	0.51	0.02
Employment	0.01	0.18	0.15	0.04	0.01	0.40	0.57	0.47	0.10	0.00
Marital status	0.30	0.10	0.79	0.95	0.63	0.58	0.28	0.99	0.89	0.74
Socioeconomic status	0.19	0.43	0.93	0.26	0.09	0.01	0.85	0.33	0.43	0.05
Household status	0.35	0.25	0.46	0.87	0.87	0.69	0.12	0.97	0.67	0.39
Surgical method	0.29	0.58	0.92	0.60	0.14	0.93	0.36	0.58	0.35	0.11
Self-management of the stoma	0.58	0.19	0.95	0.34	0.03	0.09	0.00	0.00	0.23	0.46
Participation in the making of preoperative decision on the stoma side	0.50	0.23	0.80	0.81	0.92	0.98	0.83	0.77	0.97	0.04
Gender	0.45	0.55	0.05	0.44	0.57	0.51	0.91	0.67	0.47	0.25
Avoidance of full trunk movements, e.g., trunk twists or extension	0.00	0.00	0.00	0.00	0.00	0.01	0.09	0.20	0.03	0.00
Contact dermatitis around the stoma site	0.36	0.83	0.21	0.43	0.06	0.32	0.45	0.11	0.78	0.00
Allergic dermatitis	0.15	0.31	0.95	0.28	0.57	0.04	0.21	0.31	0.10	0.01
Fungal infections of the skin around the stoma site	0.00	0.00	0.07	0.01	0.01	0.01	0.07	0.01	0.32	0.00
Pyoderma gangrenosum	0.03	0.65	1	1	1	0.23	1	1	0.09	0.05
Deep ulcerations of the skin around the stoma site	0.24	0.78	0.19	1	0.41	0.36	0.63	0.70	0.04	0.20
Bowel obstruction	0.58	0.01	0.14	0.37	0.58	0.47	0.17	0.54	0.63	0.39
Mechanical injuries	0.00	0.04	0.01	0.02	0.00	0.58	0.17	0.00	0.74	0.00
Number of complications	0.00	0.46	0.04	0.03	0.08	0.03	0.73	0.45	0.53	0.00

**Table 4:** Spearman’s correlation coefficient between the intensity of back pain as assessed by the Oswestry questionnaire on the global quality of life as self-assessed by the patients using the WHOQOL-BREF questionnaire.

	Spearman’s Correlation Coefficient (*p*-Value)
Pain intensity	−0.54 (<0.001)
Lifting	−0.19 (0.05)
Sitting	−0.13 (0.19)
Sleeping	−0.24 (0.01)
Traveling	−0.40 (<0.001)
Personal care	−0.24 (0.01)
Walking	−0.13 (0.19)
Standing	−0.12 (0.24)
Social life	−0.12 (0.21)
Change in pain intensity	−0.56 (<0.001)
Total ODI score	−0.44 (<0.001)
Number of complications	−0.78 (<0001)

**Table 5:** The results of Dunn’s test for the differences in health-related quality of life as self-assessed by the study patients using the WHOQOL-BREF according to the degree of disability.

		Degree of Disability			
		None	Mild	Moderate	Severe
Degree of disability (Dunn’s test)	**None**	1	0.51	0.00	0.01
	**Mild**	0.51	1	0.05	0.17
	**Moderate**	0.00	0.05	1	1
	**Severe**	0.01	0.17	1	1

## Data Availability

The datasets generated and/or analyzed during the current study are available from the corresponding author on reasonable request.
